# Supplementary material for: Neural correlates of memory updating in the primate prefrontal cortex
Source: Commun Biol. 2025 Jun 9;8:900. doi: 10.1038/s42003-025-08271-w (PMC12149290; doi:10.1038/s42003-025-08271-w)
Supplement: Supplementary file 2 — Description of Additional Supplementary Files [file 42003_2025_8271_MOESM2_ESM.docx]

Description of Additional Supplementary Files

**File name:** Supplementary Movie 1

**Description:** Oculomotor n-back task.

**File name:** Supplementary Data 1

**Description:** The source data underlying the graphs presented in the main figures.
