# Supplementary material for: Neural correlates of memory updating in the primate prefrontal cortex
Source: Commun Biol. 2025 Jun 9;8:900. doi: 10.1038/s42003-025-08271-w (PMC12149290; doi:10.1038/s42003-025-08271-w)
Supplement: Supplementary file 5 — Reporting Summary [file 42003_2025_8271_MOESM5_ESM.pdf]

nature portfolio

Corresponding author(s): Masaki Tanaka, Ryo Sawagashira

Last updated by author(s): Apr 29, 2025

Reporting Summary

Nature Portfolio wishes to improve the reproducibility of the work that we publish. This form provides structure for consistency and transparency in reporting. For further information on Nature Portfolio policies, see our Editorial Policies and the Editorial Policy Checklist.

Statistics

For all statistical analyses, confirm that the following items are present in the figure legend, table legend, main text, or Methods section.

n/a	Confirmed	
☐	☒	The exact sample size (*n*) for each experimental group/condition, given as a discrete number and unit of measurement
☐	☒	A statement on whether measurements were taken from distinct samples or whether the same sample was measured repeatedly
☐	☒	The statistical test(s) used AND whether they are one- or two-sided<br>*Only common tests should be described solely by name; describe more complex techniques in the Methods section.*
☐	☒	A description of all covariates tested
☐	☒	A description of any assumptions or corrections, such as tests of normality and adjustment for multiple comparisons
☐	☒	A full description of the statistical parameters including central tendency (e.g. means) or other basic estimates (e.g. regression coefficient) AND variation (e.g. standard deviation) or associated estimates of uncertainty (e.g. confidence intervals)
☐	☒	For null hypothesis testing, the test statistic (e.g. *F*, *t*, *r*) with confidence intervals, effect sizes, degrees of freedom and *P* value noted<br>*Give P values as exact values whenever suitable.*
☒	☐	For Bayesian analysis, information on the choice of priors and Markov chain Monte Carlo settings
☒	☐	For hierarchical and complex designs, identification of the appropriate level for tests and full reporting of outcomes
☒	☐	Estimates of effect sizes (e.g. Cohen's *d*, Pearson's *r*), indicating how they were calculated

Our web collection on statistics for biologists contains articles on many of the points above.

Software and code

Policy information about availability of computer code

Data collection	Omniplex (Plexon), Tempo (Reflective Computing)
Data analysis	Matlab (Mathworks)

For manuscripts utilizing custom algorithms or software that are central to the research but not yet described in published literature, software must be made available to editors and reviewers. We strongly encourage code deposition in a community repository (e.g. GitHub). See the Nature Portfolio guidelines for submitting code & software for further information.

Data

Policy information about availability of data

All manuscripts must include a data availability statement. This statement should provide the following information, where applicable:

- Accession codes, unique identifiers, or web links for publicly available datasets
- A description of any restrictions on data availability
- For clinical datasets or third party data, please ensure that the statement adheres to our policy

The data supporting the findings of this study are available from the authors upon reasonable request. All source data underlying the graphs presented in the main figures are provided in Supplementary Data.

Research involving human participants, their data, or biological material

Policy information about studies with human participants or human data. See also policy information about sex, gender (identity/presentation), and sexual orientation and race, ethnicity and racism.

Reporting on sex and gender	n/a
Reporting on race, ethnicity, or other socially relevant groupings	n/a
Population characteristics	n/a
Recruitment	n/a
Ethics oversight	n/a

Note that full information on the approval of the study protocol must also be provided in the manuscript.

Field-specific reporting

Please select the one below that is the best fit for your research. If you are not sure, read the appropriate sections before making your selection.

☒ Life sciences ☐ Behavioural & social sciences ☐ Ecological, evolutionary & environmental sciences

For a reference copy of the document with all sections, see nature.com/documents/nr-reporting-summary-flat.pdf

Life sciences study design

All studies must disclose on these points even when the disclosure is negative.

Sample size	Three male macaque monkeys were used (n = 3), and a total of 152 task-related neurons were recorded. No a priori power calculation was conducted due to the exploratory nature of the study. (Methods: Animal preparation, Analysis of neuronal data)
Data exclusions	Error trials due to fixation breaks were excluded based on a priori defined criteria. For each analysis, the exact number of animals or neurons is reported in the text or figure legends. (Methods: Analysis of behavioral and neuronal data)
Replication	Activity of single neurons was tested in multiple trials. Signals related to specific event were collected from many neurons as described in the Results. Data were collected from three behaving monkeys trained on the same behavioral tasks.
Randomization	Stimulus conditions and delay periods were randomly interleaved within sessions to reduce systematic bias. Animals were not randomly assigned to groups as all animals experienced all conditions. (Methods: Behavioral task)
Blinding	Experimenters were not blinded during task administration or analysis; however, conditions were pseudo-randomized and electrical stimulation was applied in a blinded fashion within sessions. (Methods: Electrical stimulation)

Reporting for specific materials, systems and methods

We require information from authors about some types of materials, experimental systems and methods used in many studies. Here, indicate whether each material, system or method listed is relevant to your study. If you are not sure if a list item applies to your research, read the appropriate section before selecting a response.

Materials & experimental systems

n/a	Involved in the study	
☒	☐	Antibodies
☒	☐	Eukaryotic cell lines
☒	☐	Palaeontology and archaeology
☐	☒	Animals and other organisms
☒	☐	Clinical data
☒	☐	Dual use research of concern
☒	☐	Plants

Methods

n/a	Involved in the study	
☒	☐	ChIP-seq
☒	☐	Flow cytometry
☐	☒	MRI-based neuroimaging

Animals and other research organisms

Policy information about studies involving animals; ARRIVE guidelines recommended for reporting animal research, and Sex and Gender in Research

Laboratory animals	Three adult male Japanese macaques (Macaca fuscata) aged 7.5–9.0 kg were used. Animals were sourced from the National Bio-Resource Project and monitored daily for health. (Methods: Animal preparation)
Wild animals	n/a
Reporting on sex	Male
Field-collected samples	n/a
Ethics oversight	All procedures were approved by the Hokkaido University Animal Care and Use Committee. Animal welfare was monitored according to institutional and national guidelines. (Methods: Animal preparation)

Note that full information on the approval of the study protocol must also be provided in the manuscript.

Plants

Seed stocks	n/a
Novel plant genotypes	n/a
Authentication	n/a

Magnetic resonance imaging

Experimental design

Design type	Structural MRI was taken to verify the location of the recording sites.
Design specifications	Structural MRI taken from anaesthetized animals
Behavioral performance measures	n/a

Acquisition

Imaging type(s)	3D T2WI
Field strength	3T
Sequence & imaging parameters	SPACE, Siemens
Area of acquisition	Whole brain

Diffusion MRI ☐ Used ☒ Not used

Preprocessing

Preprocessing software	n/a
Normalization	n/a
Normalization template	n/a
Noise and artifact removal	n/a
Volume censoring	n/a

Statistical modeling & inference

Model type and settings: n/a

Effect(s) tested: n/a

Specify type of analysis: ☐ Whole brain ☒ ROI-based ☐ Both

Anatomical location(s): arcuate sulcus

Statistic type for inference (See Eklund et al. 2016): n/a

Correction: n/a

Models & analysis

n/a	Involved in the study
☒	☐ Functional and/or effective connectivity
☒	☐ Graph analysis
☒	☐ Multivariate modeling or predictive analysis
